# Macrophages-Related Genes Biomarkers in the Deterioration of Atherosclerosis

**DOI:** 10.3389/fcvm.2022.890321

**Published:** 2022-06-30

**Authors:** Yue Zheng, Bingcai Qi, Wenqing Gao, Zhenchang Qi, Yanwu Liu, Yuchao Wang, Jianyu Feng, Xian Cheng, Zhiqiang Luo, Tong Li

**Affiliations:** ^1^School of Medicine, Nankai University, Tianjin, China; ^2^Department of Heart Center, The Third Central Hospital of Tianjin, Tianjin, China; ^3^Nankai University Affiliated Third Center Hospital, Tianjin, China; ^4^The Third Central Clinical College of Tianjin Medical University, Tianjin, China; ^5^Tianjin Key Laboratory of Extracorporeal Life Support for Critical Diseases, Tianjin, China; ^6^Artificial Cell Engineering Technology Research Center, Tianjin, China

**Keywords:** macrophages, PPI, differentially expressed genes, immune infiltration, GO/KEGG pathways analysis, GSEA, atherosclerosis, progression

## Abstract

**Background:**

The macrophages are involved in all stages of cardiovascular diseases, demonstrating the correlation between inflammation, atherosclerosis, and myocardial infarction (MI). Here, we aim to investigate macrophages-related genes in the deterioration of atherosclerosis.

**Methods:**

GSE41571 was downloaded and the abundance of immune cells was estimated by utilizing the xCell. By utilizing the limma test and correlation analysis, differentially expressed macrophages-related genes (DEMRGs) were documented. The functional pathways and the protein–protein interaction (PPI) network were analyzed and the hub DEMRGs were obtained. The hub DEMRGs and their interactions were analyzed using NetworkAnalyst 3.0 and for validation, the expressions of hub DEMRGs were analyzed using the GSE135055 and GSE116250 datasets as well as atherosclerosis and MI mice model.

**Results:**

A total of 509 differentially expressed genes (DEGs) were correlated with the abundance of macrophages and were identified as DEMRGs (Pearson correlation coefficients (PCC) > 0.6), which were mainly enriched in extracellular structure organization, lysosomal membrane, MHC protein complex binding, and so on. After screening out, 28 hub DEMRGs were obtained with degrees ≥20, including GNAI1 (degree = 113), MRPS2 (degree = 56), HCK (degree = 45), SOCS3 (degree = 40), NET1 (degree = 28), and so on. After validating using Gene Expression Omnibus (GEO) datasets and the atherosclerosis and MI mice model, eight proteins were validated using ApoE-/- and C57 mice. The expression levels of proteins, including SYNJ2, NET1, FZD7, LCP2, HCK, GNB2, and PPP4C were positively correlated to left ventricular ejection fraction (LVEF), while that of EIF4EBP1 was negatively correlated to LVEF.

**Conclusion:**

The screened hub DEMRGs, SYNJ2, NET1, FZD7, LCP2, HCK, GNB2, EIF4EBP1, and PPP4C, may be therapeutic targets for treatment and prediction in the patients with plaque progression and MI recurrent events. The kit of the eight hub DEMRGs may test plaque progression and MI recurrent events and help in the diagnosis and treatment of MI-induced heart failure (HF), thus decreasing mortality and morbidity.

## Introduction

Coronary artery disease (CAD) leads to considerable mortality and contributes to about one in every seven deaths in low- and middle-income countries (1). Acute myocardial infarction (MI) mortality has increased 5.6-fold in the last three decades and obesity has become the major cause of morbidity and mortality in some chronic diseases, for instance, diabetes and CAD (2). Young patients with type 2 diabetic MI develop higher long-term adverse events, suggesting that more aggressive secondary prevention is needed for those patients (2, 3). According to conservative estimates, China has 330 million people with CAD, which may result in an unacceptable burden due to recurrent cardiovascular events.

Myocardial infarction promotes atherosclerosis and thus MI progresses by releasing the progenitor cells and hematopoietic stem cells from the bone marrow niche, further increasing the number of monocytes (4, 5). The vasomotor function of the endothelium can be consistently impaired when patients develop ST-segment elevation myocardial infarction (STEMI), which is highly correlated to atherosclerosis development and plaque progression (6). Therefore, biomarkers associated with plaque progression should be investigated for coronary disease progression and MI recurrence, which may help decrease the adverse cardiovascular events.

Macrophages participate in all stages of CAD, suggesting the correlation between inflammation and atherosclerosis (7, 8). The migration of macrophages populating the plaques decreased, resulting in inflammation-resolved failure and further complicated atherosclerotic plaque (9). However, the correlations between these biomarkers of macrophages and molecular mechanisms in atherosclerosis and MI recurrence are still unclear.

In this study, the abundance of macrophages in plaques was analyzed using immune infiltration analysis. The differentially expressed macrophages-related genes (DEMRGs) between ruptured and stable plaques were investigated and the hub genes were explored and validated utilizing Gene Expression Omnibus (GEO) datasets and mice model, which may provide potential targets for plaque progression and adverse cardiovascular events.

## Methods

### Data Source

Using the keywords “atherosclerosis” and “Homo sapiens”, GSE41571 from the GEO was downloaded (10). There were five ruptured plaques and six stable plaques based on the Affymetrix Human Genome U133 Plus 2.0 Array. At first, raw transcriptomic data were processed with log2 transformation utilizing a robust multichip average algorithm (Supplementary Figure S1) (11).

### Evaluation of Immune Cells

xCell can digitally portray the tissue cellular heterogeneity landscape (12). To obtain the abundance of stroma and immune cells in the plaques, xCell analysis was utilized in the GSE41571 dataset.

### Identification of DEMRGs

The differentially expressed genes (DEGs) were explored by utilizing the “limma” package. Log2|fold change|>1 and *p* < 0.05 were the thresholds. Then, Pearson correlation analysis was applied to obtain the DEGs related to macrophages. The DEGs with Pearson correlation coefficients (PCC) >0.6 were regarded as DEMRGs.

### Enrichment Analysis

To understand the functions of DEMRGs, Gene Ontology (GO)/Kyoto Encyclopedia of Genes and Genomes (KEGG) pathway analysis, as well as Gene Set Enrichment Analysis (GSEA) analysis, were applied utilizing the “clusterProfiler” package in R (13). A *p* < 0.05 was set as the significance threshold for enrichment analyses.

### Protein–Protein Interaction (PPI) Network Analysis

NetworkAnalyst 3.0 is a comprehensive network visual analyzed platform for gene expression analysis (14). To explore the hub genes, DEMRGs were uploaded to NetworkAnalyst 3.0 to investigate the protein network interaction diagram and significant PPIs were identified with a combined score of >0.4. The nodes with a connectivity degree ≥20 were regarded as the hub DEMRGs. GO and KEGG pathway analysis was also utilized to understand the functions of hub DEMRGs.

### The Hub DEMRGs and Their Interactions

The hub DEMRGs and their interactions were analyzed using NetworkAnalyst 3.0. Specifically, transcription factors (TFs)-miRNA coregulatory interactions with five screened hub DEMRGs were shown using the RegNetwork repository where the literature curated regulatory interaction information was collected (applicable for human and mouse data only). Left ventricle tissue-specific PPIs were shown using the DifferentialNet database (Filter is 15), which shows the differential PPIs across human tissues. The hub DEMRGs-chemical interactions were shown using the Comparative Toxicogenomics Database (CTD). The hub DEMRGs-drugs interactions were shown using the DrugBank database (Version 5.0). The hub DEMRGs were also validated in THP-1 cells from the Immuno-Navigator database.

### Validation of the Hub DEMRGs Using GEO Datasets

To explore the correlation between the hub DEMRGs and plaque progression, we performed the GSE163154 dataset to investigate the expressions of the hub DEMRGs in plaques with or without intraplaque hemorrhage (15). Then, the expression patterns of the screened DEMRGs in the left ventricular remodeling process induced by heart failure were validated in the independent datasets, including GSE135055 (*n* = 30) (16) and GSE116250 (*n* = 64) (17, 18). The information is shown in Supplementary Table S1.

Receiver operation characteristic (ROC) curve and joint ROC curve analyses were conducted to investigate the diagnostic value of the DEMRGs and the area under the curve (AUC) was determined using the “pROC” package.

### Validation of the Hub DEMRGs Using Mice Models

To further validate the hub DEMRGs effects, atherosclerosis and MI mice models were utilized. Adult experimental C57Bl/6J and ApoE^−/−^ male mice were purchased (Charles River). Mice were maintained in an specific pathogen free (SPF) environment (temperature: 23–25°C; humidity: 55–60%) with free access to food and water and a 12/12 light-dark cycle. The protocols were approved by the Institute of Radiation Medicine, the Chinese Academy of Medical Science, China, which conform to the Guide for the Care and Use of Laboratory Animals.

To construct the atherosclerosis model, the ApoE^−/−^ male mice (n = 6 per group) were fed a Western diet for 6 months (HFHC100244). To construct the MI model, the ApoE^−/−^ (*n* = 7 per group) and C57Bl/6J (*n* = 6 per group) male mice were fed with the Western diet for 6 months and the MI mice model was constructed with left coronary descending ligation through gaseous anesthesia (1.5–2% isoflurane, MSS-3, England). Sham animals underwent the same procedure without ligation. To reduce mice pain in animal experiments, the animals were euthanized by cervical dislocation after isoflurane anesthesia (5%, MSS-3, England).

### Echocardiographic Examination

Cardiac function was evaluated using the Vevo® 2100 System equipped with a 30 MHz transducer (FUJIFILM VisualSonics, Inc. Toronto, Canada). The investigator was blinded to group assignment. Mice were anesthetized through inhalation of isoflurane (1–1.5%, MSS-3, England). Left ventricular ejection fraction (LVEF, %) and left ventricular fraction shortening (LVFS, %) of mice in the atherosclerosis and MI model 2 days after MI were measured with M-mode through a two-dimensional parasternal long axis.

### Enzyme-Linked Immunosorbent Assay

In the atherosclerosis mice model, the aorta root was collected from ApoE-/- mice and was control fed with high fat diet. In the MI mice model, the infarcted area and border zones were collected together in MI mice, and the same areas in SHAM mice were also collected. The proteins were extracted according to the kit instruction (BC3710, Solarbio). For further validations, the protein level of SYNJ2, NET1, FZD7, TBL1XR1, GNB2, PPP4C, EIF4EBP1, HCK, and LCP2 were explored with the 4°Cincubation overnight of mouse SYNJ2 primary antibodies (1:1,000, 13893-1-AP, Proteintech), mouse NET1 primary antibodies (1:500, 28180-1-AP, Proteintech), mouse FZD7 primary antibodies (1:500, 16974-1-AP, Proteintech), mouse TBL1XR1 primary antibodies (1:1,000, 55312-1-AP, Proteintech), mouse GNB2 primary antibodies (1:500, 16090-1-AP, Proteintech), mouse PPP4C primary antibodies (1:250, 10262-1-AP, Proteintech), mouse EIF4EBP1 primary antibodies (1:500, 60246-1-Ig, Proteintech), mouse HCK primary antibodies (1:500, 11600-1-AP, Proteintech), and mouse LCP2 primary antibodies (1:500, 12728-1-AP, Proteintech), respectively. The serum matrix (MILLIPLEX Analyst Kit, Millipore) was used as the positive control in the validation.

### Statistical Analysis

All data are presented as the mean ± SD. Statistical analyses were performed using SPSS 23.0. The Shapiro–Wilk normality test and Welch's *t*-test were used and the Spearman correlation analysis was applied among LVEF and protein levels of screened hub DEMRGs. A value of *p* < 0.05 was considered statistically significant.

## Results

### DEMRGs in Plaque Progression

**Figure 1:**
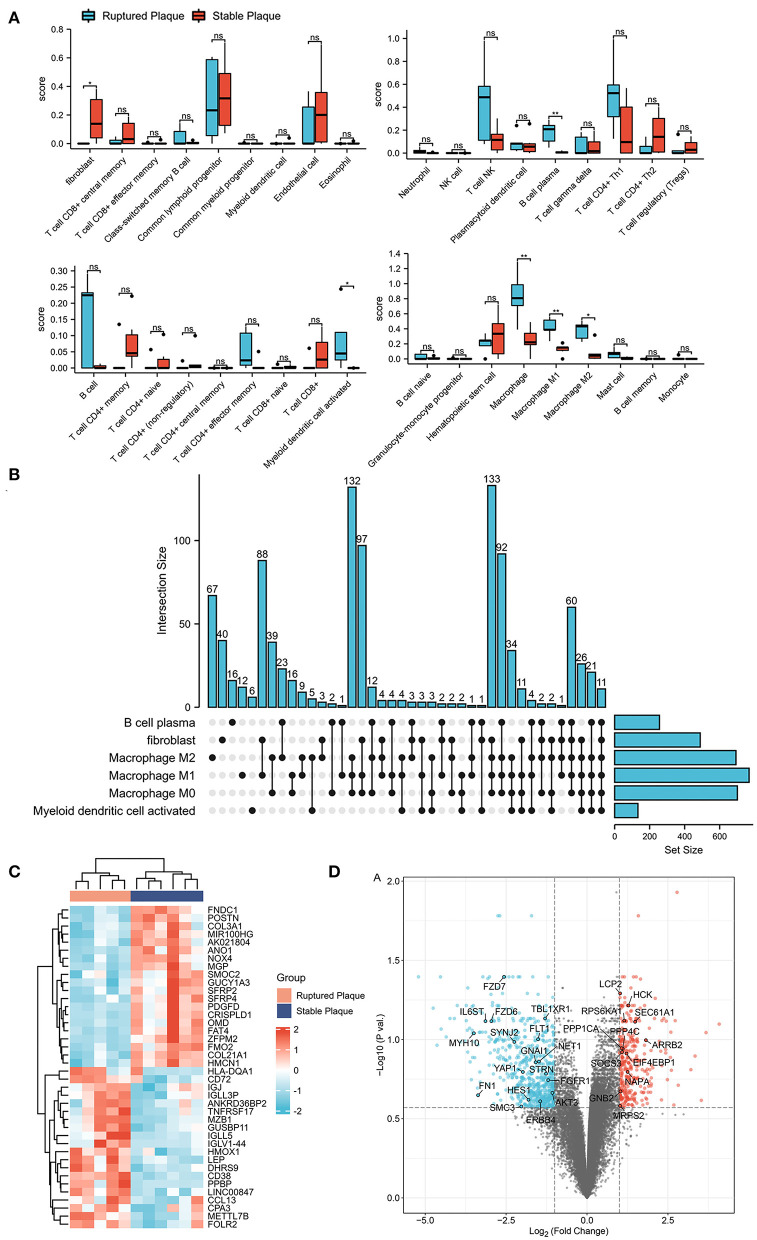
Identification of differentially expressed genes (DEGs) in ruptured plaques and stable plaques. **(A)** Abundance of immune cells estimated by the xCell algorithm in GSE41571. **(B)** Upset diagram of plasm B cells, fibroblasts, activated myeloid dendritic cells, M0, M1, and M2 Macrophages-related DEGs with Pearson correlation coefficients >0.6. **(C,D)** Heatmap **(C)** and Volcano plot **(D)** of DEGs between ruptured plaques and stable plaques.

**Figure 2:**
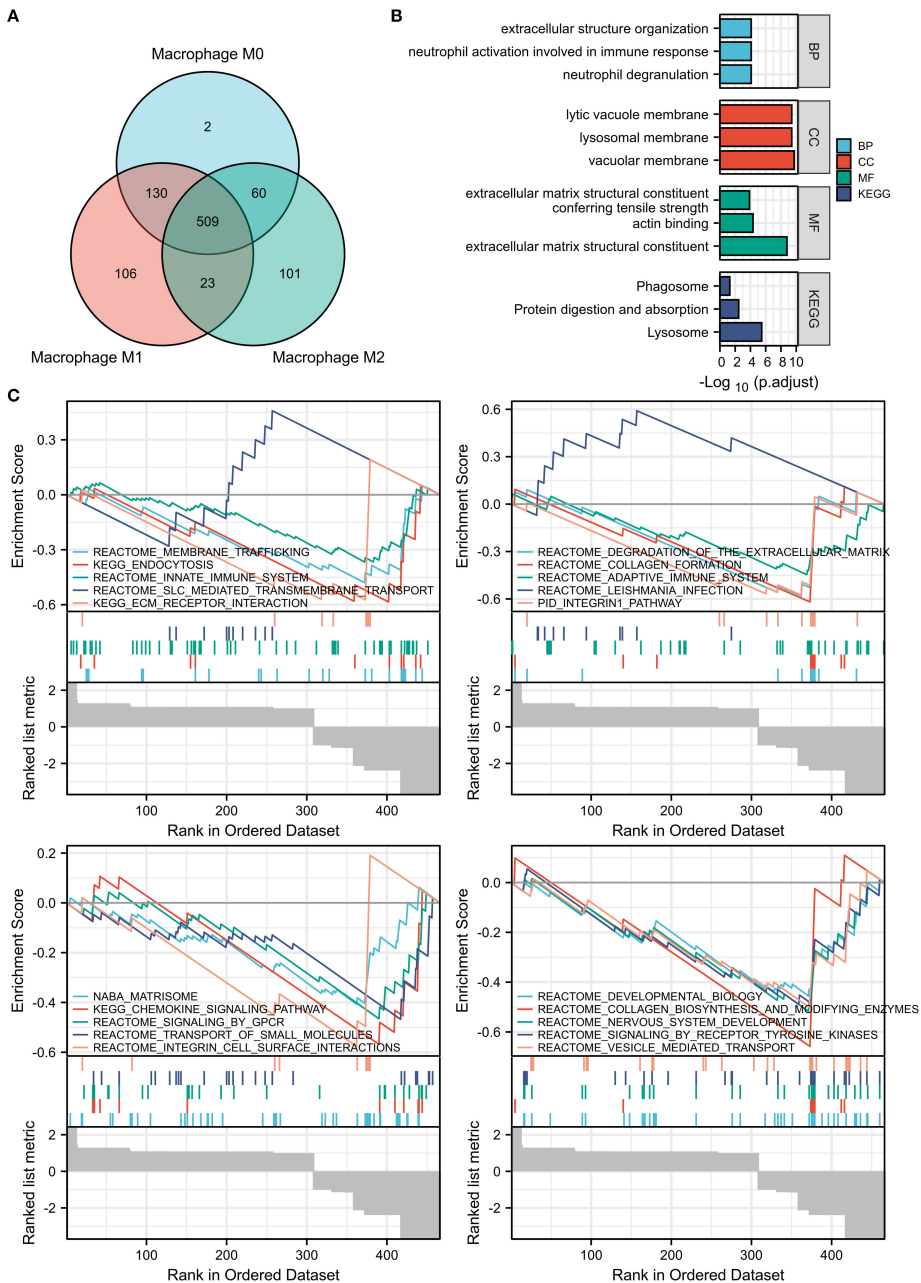
Enrichment pathway analysis of the DEMRGs. **(A)** Venn diagram showed 509 differentially expressed genes (DEGs) among M0, M1, and M2 macrophages were regarded as DEMRGs. **(B)** The significant Gene Ontology (GO) and Kyoto Encyclopedia of Genes and Genomes (KEGG) pathways enriched by DEMRGs. **(C)** The gene sets associated with DEMRGs using Gene Set Enrichment Analysis (GSEA) analysis. DEMRGs, differentially expressed macrophages-related genes.

The xCell analysis was applied to obtain the immune cell abundance in the GSE41571 dataset. The plaques in the ruptured stage demonstrated a higher infiltration of plasma B cells, myeloid-activated dendritic cells, and macrophages (Figure 1A; Supplementary Figure S2). A total of 1,043 DEGs were obtained and out of these, 701 were highly correlated (PCC > 0.6) with the abundance of M0 macrophages, 768 were highly correlated (PCC > 0.6) with the abundance of M1 macrophages, and 693 were highly correlated (PCC > 0.6) with the abundance of M2 macrophages (Figures 1B–D). It was found that M0 macrophages are unpolarized; M1 macrophages are pro-inflammatory, and; M2 macrophages are anti-inflammatory, and a Venn diagram was used to identify the same DEGs among M0, M1, and M2 macrophages out of which 509 DEGs were identified as DEMRGs (Figure 2A).

### Functional Enrichment for DEMRGs

The list of DEMRGs was uploaded to the Xiantao web page (www.xiantao.love) for functional analysis, which was involved in extracellular structure organization, lysosomal membrane, and MHC protein complex binding. Besides, KEGG enrichment analysis demonstrated that the pathways correlated to DEMRGs mainly included lysosome, protein digestion and absorption, phagosome, and toxoplasmosis (Figure 2B; Supplementary Figure S3; Table 1). GSEA analysis showed that DEMRGs mainly included REACTOME_DEVELOPMENTAL_BIOLOGY, NABA_MATRISOME, and REACTOME_SIGNALING_BY_RECEPTOR_TYROSINE_KINASES (Table 2). Interestingly, the normalized enrichment score (NES) of REACTOME_LEISHMANIA_INFECTION and REACTOME_SLC_MEDIATED_TRANSMEMBRANE_TRANSPORT were 2.117895264 and 1.643584603, respectively, while other NES were negative (Figure 2C).

**Table 1:** The significant GO and KEGG pathways enriched by 509 DEGs.

**GO**	**ID**	**Description**	**GeneRatio**	**BgRatio**	***p*-value**	***p*.adjust**	***q*-value**
BP	GO:0043312	neutrophil degranulation	33/438	485/18670	4.91e-08	7.95e-05	6.90e-05
BP	GO:0002283	neutrophil activation involved in immune response	33/438	488/18670	5.68e-08	7.95e-05	6.90e-05
BP	GO:0043062	extracellular structure organization	30/438	422/18670	7.82e-08	7.95e-05	6.90e-05
BP	GO:0042119	neutrophil activation	33/438	498/18670	9.14e-08	7.95e-05	6.90e-05
BP	GO:0002446	neutrophil mediated immunity	33/438	499/18670	9.58e-08	7.95e-05	6.90e-05
CC	GO:0005774	vacuolar membrane	38/455	412/19717	3.97e-13	1.83e-10	1.52e-10
CC	GO:0005765	lysosomal membrane	34/455	354/19717	2.31e-12	3.84e-10	3.20e-10
CC	GO:0098852	lytic vacuole membrane	34/455	355/19717	2.50e-12	3.84e-10	3.20e-10
CC	GO:0062023	collagen-containing extracellular matrix	35/455	406/19717	2.36e-11	2.71e-09	2.26e-09
CC	GO:0005581	collagen trimer	14/455	87/19717	1.16e-08	1.07e-06	8.87e-07
MF	GO:0005201	extracellular matrix structural constituent	24/441	163/17697	2.45e-12	1.64e-09	1.56e-09
MF	GO:0003779	actin binding	31/441	431/17697	1.37e-07	4.59e-05	4.38e-05
MF	GO:0030020	extracellular matrix structural constituent conferring tensile strength	9/441	41/17697	5.90e-07	1.32e-04	1.26e-04
MF	GO:0050839	cell adhesion molecule binding	30/441	499/17697	8.67e-06	0.001	0.001
MF	GO:0023023	MHC protein complex binding	6/441	25/17697	2.74e-05	0.004	0.003
KEGG	hsa04142	Lysosome	18/224	128/8076	1.21e-08	3.31e-06	3.04e-06
KEGG	hsa04974	Protein digestion and absorption	12/224	103/8076	2.55e-05	0.003	0.003
KEGG	hsa04145	Phagosome	12/224	152/8076	0.001	0.050	0.046
KEGG	hsa05145	Toxoplasmosis	10/224	112/8076	0.001	0.050	0.046
KEGG	hsa04940	Type I diabetes mellitus	6/224	43/8076	0.001	0.050	0.046

*DEGs, Different Expressed Genes; GO, Gene ONTOLOGY; BP, Biological Process; CC, cellular component; MF, Molecular Function; KEGG, Kyoto Encyclopedia of Genes and Genomes*.

**Table 2:** The gene set enrichment analysis of 509 DEGs.

**ID**	**Setsize**	**EnrichmentScore**	**NES**	***p*-value**	***p*.adjust**	***q*-values**	**Rank**
REACTOME_DEVELOPMENTAL_BIOLOGY	42	−0.455736963	−1.810929732	0.003215434	0.077020202	0.054492291	96
REACTOME_COLLAGEN_BIOSYNTHESIS_AND_MODIFYING_ENZYMES	10	−0.658049427	−1.851927505	0.003968254	0.077020202	0.054492291	94
REACTOME_NERVOUS_SYSTEM_DEVELOPMENT	29	−0.516762957	−1.911346319	0.004424779	0.077020202	0.054492291	96
REACTOME_SIGNALING_BY_RECEPTOR_TYROSINE_KINASES	29	−0.48960486	−1.81089692	0.009955752	0.077020202	0.054492291	108
REACTOME_VESICLE_MEDIATED_TRANSPORT	27	−0.5008811	−1.816707985	0.011261261	0.077020202	0.054492291	95
REACTOME_DEGRADATION_OF_THE_EXTRACELLULAR_MATRIX	13	−0.581448267	−1.771341344	0.011363636	0.077020202	0.054492291	105
REACTOME_COLLAGEN_FORMATION	11	−0.618867217	−1.789243746	0.011673152	0.077020202	0.054492291	94
REACTOME_ADAPTIVE_IMMUNE_SYSTEM	38	−0.446518499	−1.740047653	0.011866235	0.077020202	0.054492291	98
REACTOME_LEISHMANIA_INFECTION	10	0.590600566	2.117895264	0.012145749	0.077020202	0.054492291	157
PID_INTEGRIN1_PATHWAY	13	−0.575607133	−1.753546738	0.012626263	0.077020202	0.054492291	105
NABA_MATRISOME	47	−0.406963953	−1.643327996	0.016985138	0.094190311	0.066640254	105
KEGG_CHEMOKINE_SIGNALING_PATHWAY	12	−0.568058265	−1.685702571	0.023017903	0.108313156	0.076632259	77
REACTOME_SIGNALING_BY_GPCR	27	−0.465333042	−1.687774311	0.023648649	0.108313156	0.076632259	77
REACTOME_TRANSPORT_OF_SMALL_MOLECULES	25	−0.467801517	−1.67862867	0.024858757	0.108313156	0.076632259	50
REACTOME_INTEGRIN_CELL_SURFACE_INTERACTIONS	11	−0.57654683	−1.666888763	0.028534371	0.110060624	0.077868603	105
REACTOME_MEMBRANE_TRAFFICKING	22	−0.479364201	−1.661487991	0.02886836	0.110060624	0.077868603	95
KEGG_ENDOCYTOSIS	10	−0.584690143	−1.645474815	0.035714286	0.128151261	0.090667846	65
REACTOME_INNATE_IMMUNE_SYSTEM	53	−0.367172397	−1.508970204	0.04	0.135555556	0.095906433	95
REACTOME_SLC_MEDIATED_TRANSMEMBRANE_TRANSPORT	10	0.458333333	1.643584603	0.044534413	0.137169312	0.097048176	257
KEGG_ECM_RECEPTOR_INTERACTION	10	−0.57270396	−1.611742482	0.044973545	0.137169312	0.097048176	94

*NES, normalized enrichment score*.

### PPI Network Analysis

**Figure 3:**
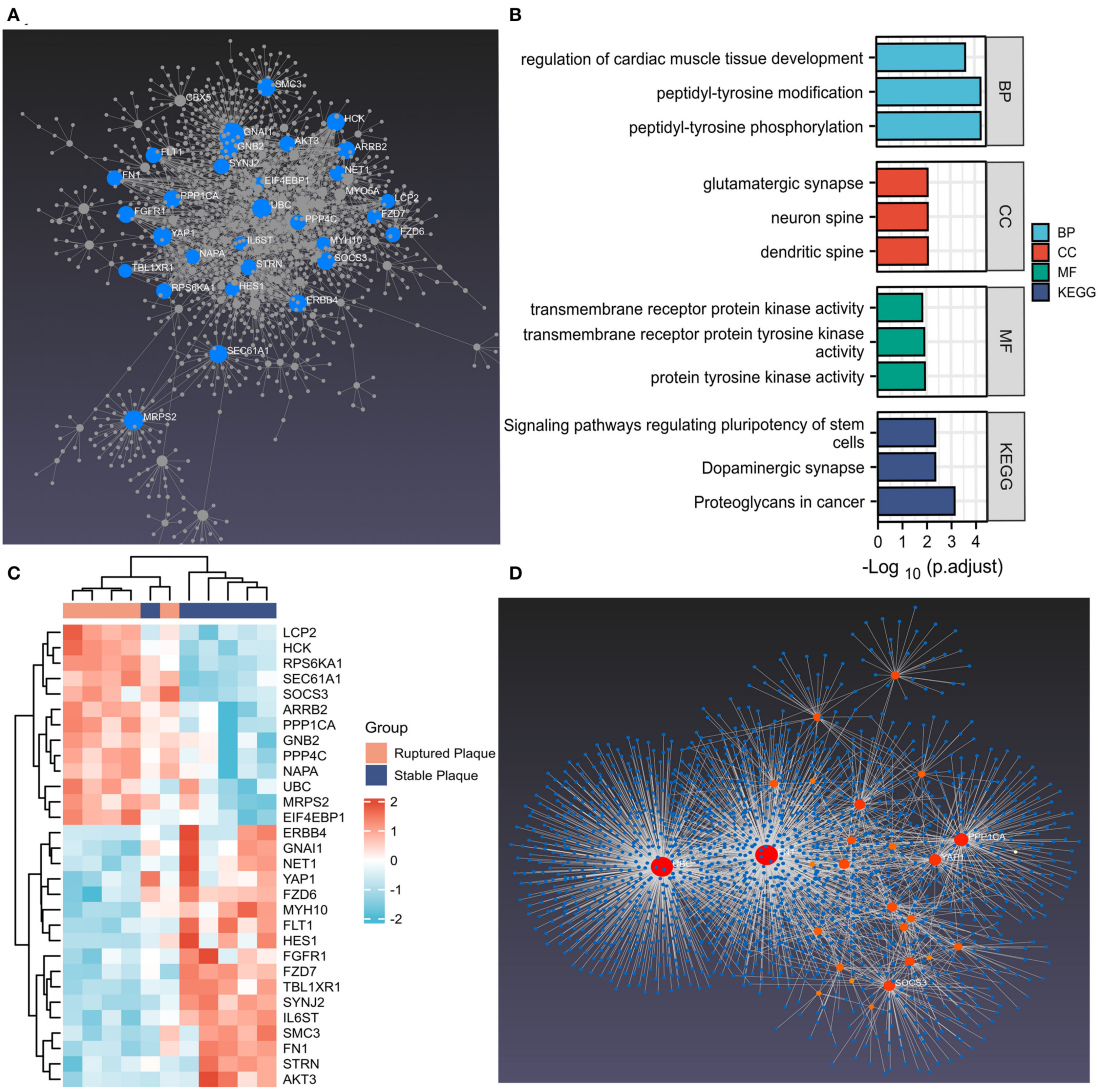
The hub differentially expressed macrophages-related genes (DEMRGs) and enrichment pathway analysis. **(A)** The hub DEMRGs were shown using NetworkAnalyst 3.0. **(B)** The significant GO and Kyoto Encyclopedia of Genes and Genomes (KEGG) pathways enriched by the hub DEMRGs. **(C)** The heatmap of the hub DEMRGs in GSE41571. **(D)** Left ventricle tissue-specific protein–protein interactions (PPIs) were applied, demonstrating UBC, FN1, PPP1CA, YAP1, and SOCS3 were core regulators in the network.

The PPI network of DEMRGs was constructed utilizing the NetworkAnalyst 3.0, which demonstrated 28 hub genes (Figure 3A; Supplementary Table S2). The DEMRGs with connective degrees ≥20 were considered as hub DEMRGs in the network, including GNAI1 (degree = 113), MRPS2 (degree = 56), HCK (degree = 45), SOCS3 (degree = 40), NET1 (degree = 28) and, etc. These genes were involved in GO such as regulation of cardiac muscle tissue development, membrane microdomain, transmembrane receptor protein kinase activity, peptidyl-tyrosine modification, and Wnt-activated receptor activity. Besides, KEGG enrichment analysis demonstrated that the signaling pathways correlated to the hub DEMRGs were mainly involved in Dopaminergic synapse, and PI3K-Akt signaling pathway (Figures 3B,C; Supplementary Figure S4; Table 3).

**Table 3:** The significant GO and KEGG pathways enriched by hub DEMRGs.

**GO**	**ID**	**Description**	**GeneRatio**	**BgRatio**	***p*-value**	**p.adjust**	***q*-value**
BP	GO:0018108	Peptidyl-tyrosine phosphorylation	8/30	363/18670	7.62e-08	5.68e-05	3.51e-05
BP	GO:0018212	Peptidyl-tyrosine modification	8/30	366/18670	8.11e-08	5.68e-05	3.51e-05
BP	GO:0055024	Regulation of cardiac muscle tissue development	5/30	100/18670	5.11e-07	2.38e-04	1.47e-04
BP	GO:0048738	Cardiac muscle tissue development	6/30	233/18670	1.64e-06	5.73e-04	3.55e-04
BP	GO:0055025	Positive regulation of cardiac muscle tissue development	4/30	62/18670	2.83e-06	6.83e-04	4.23e-04
CC	GO:0043197	Dendritic spine	4/30	169/19717	1.20e-04	0.008	0.006
CC	GO:0044309	Neuron spine	4/30	171/19717	1.26e-04	0.008	0.006
CC	GO:0098978	Glutamatergic synapse	5/30	349/19717	1.67e-04	0.008	0.006
CC	GO:0045121	Membrane raft	4/30	315/19717	0.001	0.037	0.027
CC	GO:0098857	Membrane microdomain	4/30	316/19717	0.001	0.037	0.027
MF	GO:0004713	Protein tyrosine kinase activity	4/30	134/17697	7.39e-05	0.012	0.008
MF	GO:0004714	Transmembrane receptor protein tyrosine kinase activity	3/30	62/17697	1.55e-04	0.012	0.008
MF	GO:0019199	Transmembrane Receptor Protein Kinase Activity	3/30	79/17697	3.19e-04	0.015	0.010
MF	GO:0042813	Wnt-activated receptor activity	2/30	17/17697	3.72e-04	0.015	0.010
MF	GO:0051721	Protein phosphatase 2A binding	2/30	32/17697	0.001	0.035	0.023
KEGG	hsa05205	Proteoglycans in cancer	7/28	205/8076	4.61e-06	7.43e-04	4.71e-04
KEGG	hsa04728	Dopaminergic synapse	5/28	132/8076	7.86e-05	0.005	0.003
KEGG	hsa04550	Signaling pathways regulating pluripotency of stem cells	5/28	143/8076	1.15e-04	0.005	0.003
KEGG	hsa05224	Breast cancer	5/28	147/8076	1.31e-04	0.005	0.003
KEGG	hsa04151	PI3K-Akt signaling pathway	7/28	354/8076	1.56e-04	0.005	0.003

*DEMRGs, Different Expressed Macrophages-related Genes; GO, Gene ONTOLOGY; BP, Biological Process; CC, cellular component; MF, Molecular Function; KEGG, Kyoto Encyclopedia of Genes andGenomes*.

### The Hub DEMRGs and Their Interactions

**Figure 4:**
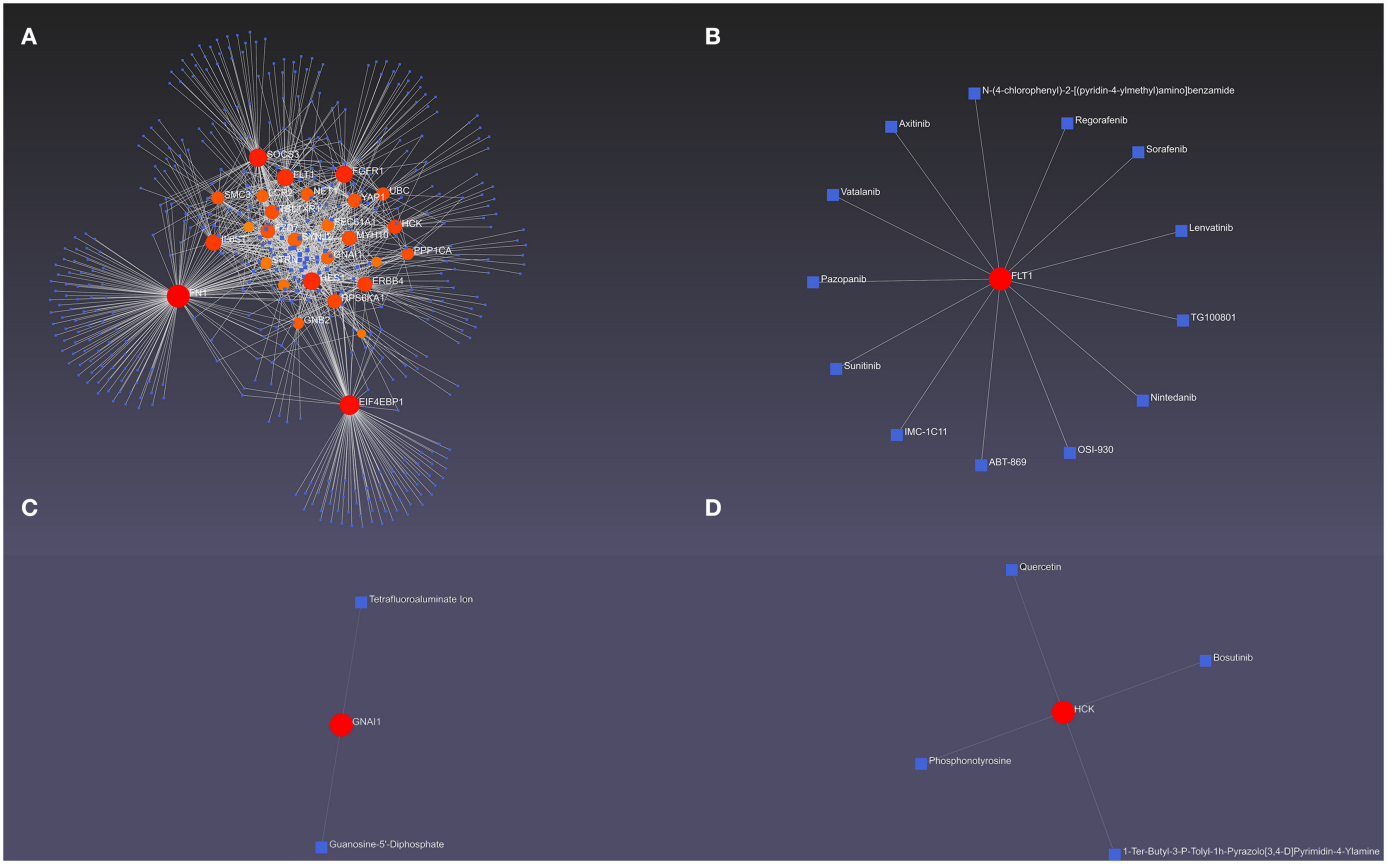
The hub differentially expressed macrophages-related genes (DEMRGs) and their interactions. **(A)** The hub DEMRGs-chemical interactions. **(B–D)** The hub DEMRGs, including FLT1 **(B)**, GNAl1 **(C)**, and HCK **(D)** and their drug interactions.

The hub DEMRGs and their interactions were analyzed using NetworkAnalyst 3.0. Left ventricle tissue-specific PPI demonstrated UBC, FN1, PPP1CA, YAP1, SOCS3 were core regulators in the network (Figure 3D). TFs-miRNA coregulatory interactions with hub DEMRGs and THP-1 cell-specific PPI demonstrated that SOCS3, LCP2, STRN, and TBL1XR1 were hub genes in the network (Supplementary Figure S5). The hub DEMRGs-chemical interactions and the hub DEMRGs-drugs interactions were also analyzed, which demonstrated the chemicals and drugs of the targets, for instance, GNAI1, FLT1, and HCK that can be used to impede plaques progression and prevent MI recurrent events (Figure 4).

### Validations of Hub DEMRGs Using GEO Datasets

**Figure 5:**
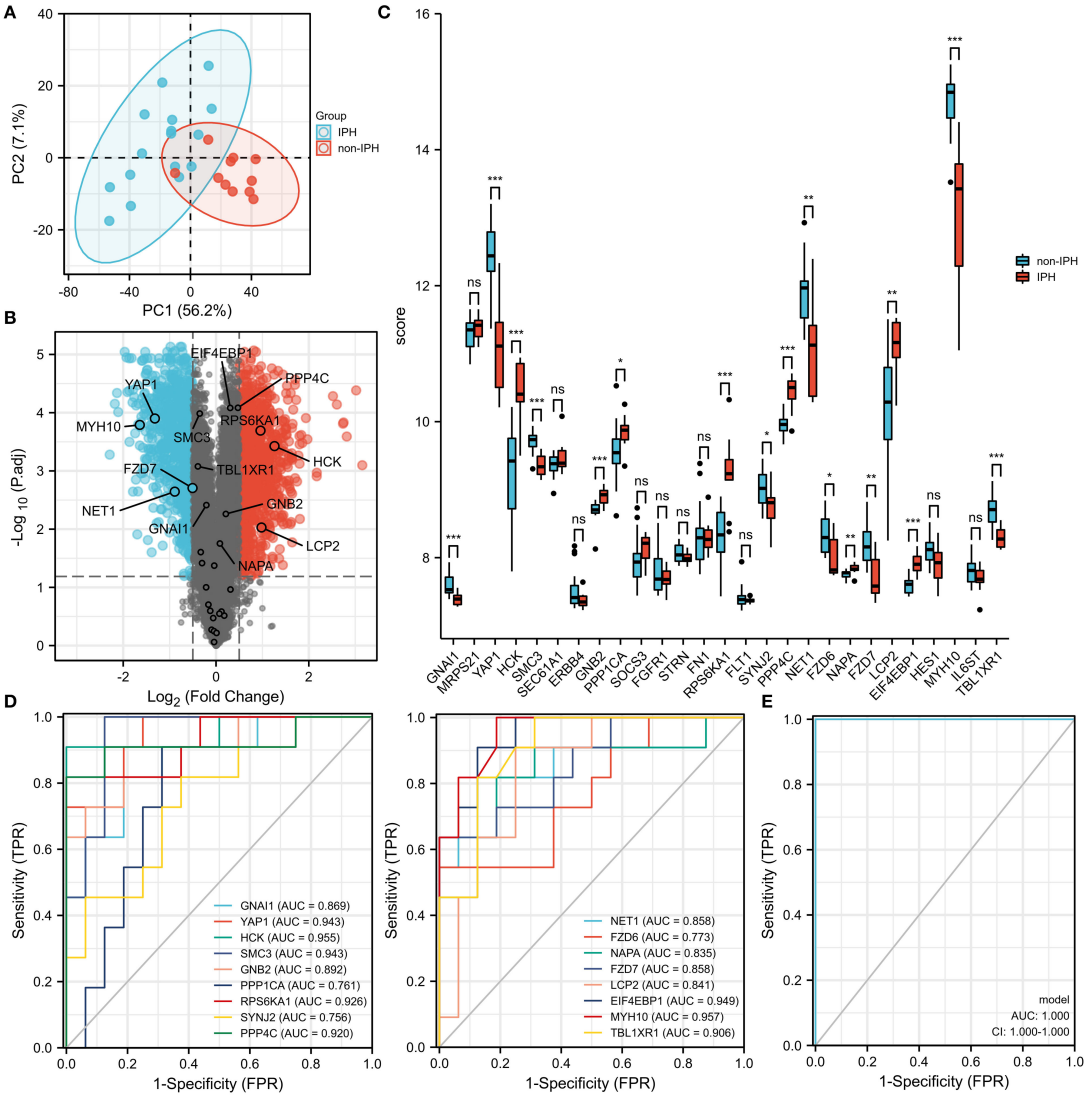
Expression patterns of the hub differentially expressed macrophages-related genes (DEMRGs) were validated in the GSE163154 dataset. **(A,B)** The principal components analysis (PCA) diagram **(A)** and Volcano plot **(B)** between the patients with intraplaque hemorrhage and non-intraplaque hemorrhage. **(C)** The expression level of the hub DEMRGs. **(D)** The receiver operation characteristic (ROC) curves of the hub DEMRGs validated in the patients with intraplaque hemorrhage compared to control. **(E)** The joint ROC curves of the hub validated DEMRGs. IPH, intraplaque hemorrhage. *p < 0.05; **p < 0.01; ***p < 0.001; ns, not significant.

To investigate the function of screened hub DEMRGs, the hub DEMRGs expression patterns were validated in the GSE163154 dataset. Compared to non-intraplaque hemorrhage patients, the patients with intraplaque hemorrhage had higher expressions of the proteins including HCK, GNB2, PPP1CA, RPS6KA1, PPP4C, NAPA, LCP2, and EIF4EBP1, while the patients had lower expressions of the proteins, such as GNAl1, YAP1, SMC3, SYNJ2, NET1, FZD6, FZD7, IL6ST, and TBL1XR1 (Figures 5A–C). ROC analysis also showed that these DEMRGs may act as diagnostic biomarkers for patients with intraplaque hemorrhage. Intriguingly, the joint ROC of the 17 DEMRGs was 1 (Figures 5D,E).

**Figure 6:**
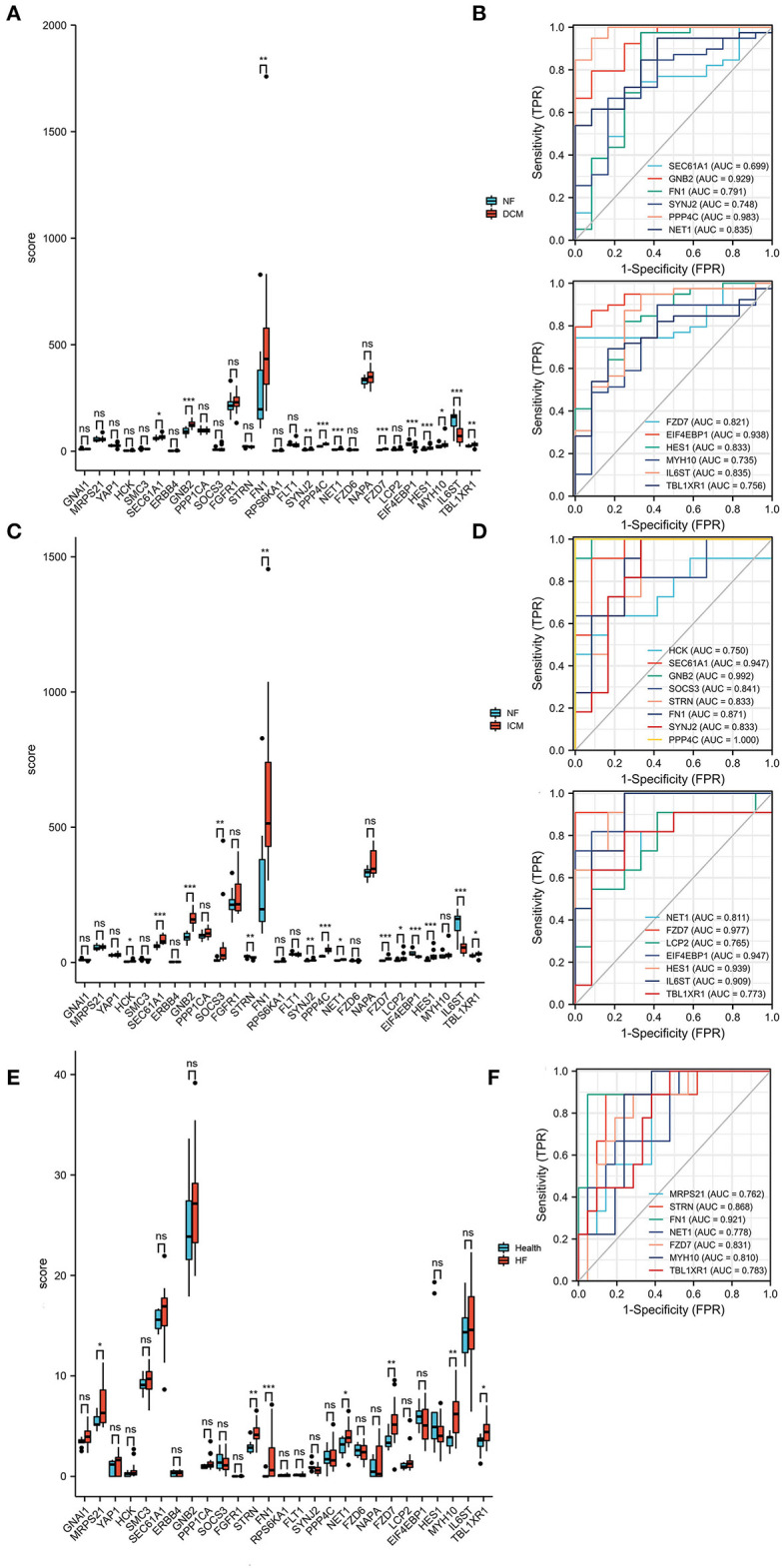
Expression patterns of the hub differentially expressed macrophages-related genes (DEMRGs) were validated in the GSE116250 and GSE135055 datasets. **(A)** The expression level of the hub DEMRGs in patients with dilated cardiomyopathy compared to control. **(B)** The receiver operation characteristic (ROC) curves of the validated DEMRGs in patients with dilated cardiomyopathy. **(C)** The expression level of the hub DEMRGs in patients with ischemic cardiomyopathy compared to control. **(D)** The ROC curves of the validated DEMRGs in patients with ischemic cardiomyopathy. **(E)** The expression level of the hub DEMRGs in patients with ischemia-induced heart failure compared to control. **(F)** The ROC curves of the validated DEMRGs in patients ischemia-induced heart failure. DCM, dilated cardiomyopathy; ICM, ischemic cardiomyopathy; HF, heart failure. *p < 0.05; **p < 0.01; ***p < 0.001; ns, not significant.

Next, the DEMRGs expression patterns were validated in two independent datasets (GSE116250 (*n* = 64) and GSE135055 (*n* = 30)). In the GSE116250 dataset, SEC61A1, GNB2, FN1, SYNJ2, PPP4C, NET1, FZD7, HES1, MYH10, and TBL1XR1 were highly expressed and EIF4EBP1 and IL6ST were lowly expressed in patients with dilated cardiomyopathy (Figure 6A). HCK, SEC61A1, GNB2, SOCS3, FN1, SYNJ2, PPP4C, NET1, FZD7, LCP2, HES1, and TBL1XR1 were highly expressed, and STRN, EIF4EBP1, and IL6ST were lowly expressed in patients with ischemic cardiomyopathy (Figure 6C). The ROC curves demonstrated that the hub DEMRGs mentioned above may act as diagnostic biomarkers for HF patients with dilated and ischemic cardiomyopathy (Figures 6B,D). To further validate this finding, the associations between the DEMRGs and HF were examined in the GSE135055 dataset, which demonstrated that the seven DEMRGs including MRPS21, STRN, FN1, NET1, FZD7, MYH10, and TBL1XR1 were all highly expressed in patients with heart failure and may act as diagnostic biomarkers for HF patients as well (Figures 6E,F).

### Validations of Hub DEMRGs Using Atherosclerosis and MI Mice Model

**Figure 7:**
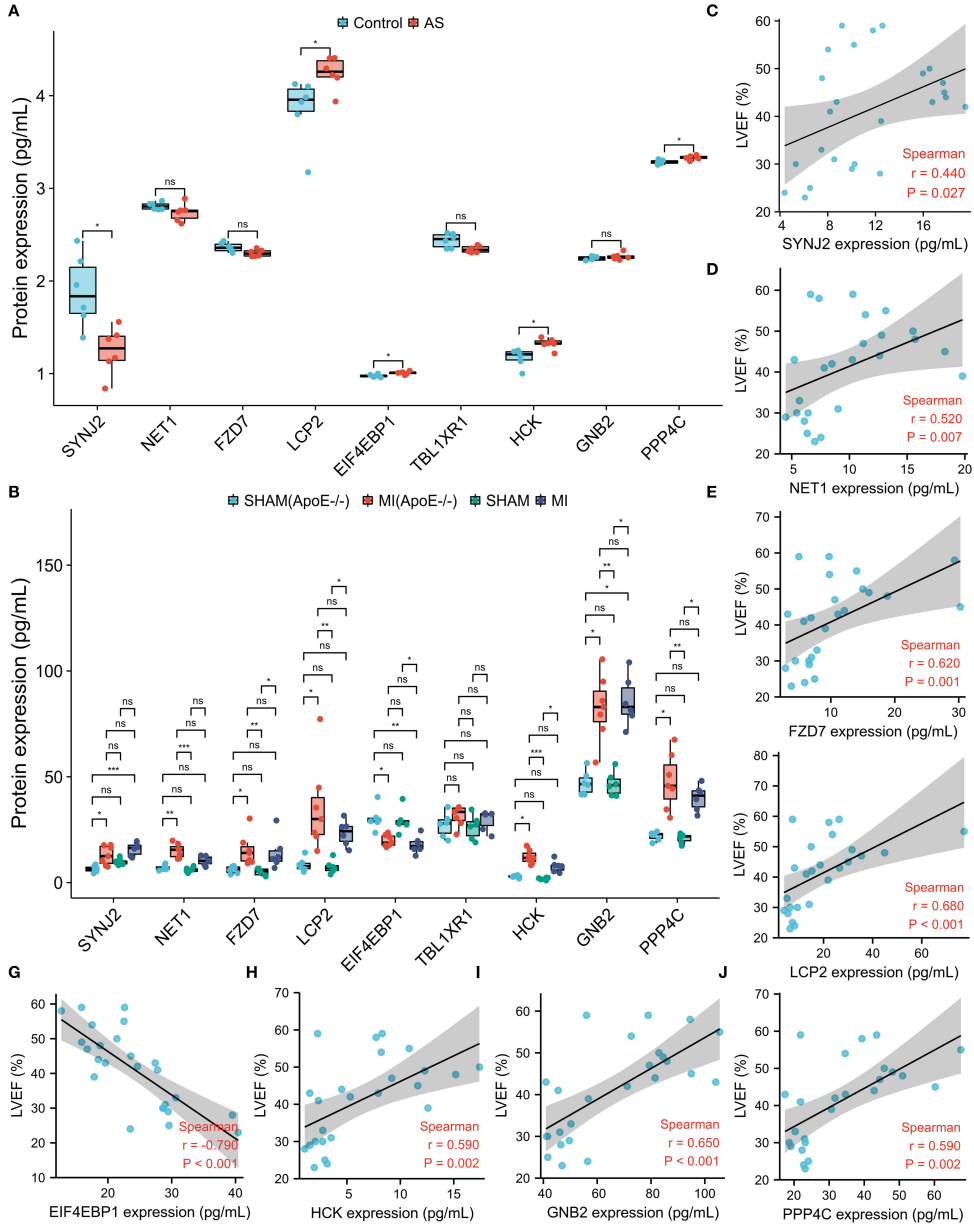
The protein expression level of the nine screened hub differentially expressed macrophages-related genes (DEMRGs) in atherosclerosis and myocardial infarction (MI) mice model. **(A)** The expression level of the nine hub DEMRGs in atherosclerosis. **(B)** The expression level of the nine hub DEMRGs in MI and SHAM groups in ApoE-/- and C57Bl/6J mice. **(C–J)** The correlation between left ventricular ejection fraction (LVEF) and the nine DEMRGs expression level in the MI mice model, including SYNJ2 **(C)**, NET1 **(D)**, FZD7 **(E)**, LCP2 **(F)**, EIF4EBP1 **(G)**, HCK **(H)**, GNB2 **(I)**, and PPP4C **(J)**. *p < 0.05; **p < 0.01; ***p < 0.001; ns, not significant.

Using the Venn diagram, the nine proteins, for instance, SYNJ2, NET1, FZD7, TBL1XR1, GNB2, PPP4C, EIF4EBP1, HCK, and LCP2 were differentially expressed in intraplaque hemorrhage and ischemic cardiomyopathy GEO datasets (Supplementary Figure S6; Table 4). To validate the correlation between the hub DEMRGs and atherosclerosis, the atherosclerosis mice model was applied and ELISA was utilized to investigate the 9 proteins expression. LCP2, EIF4EBP1, HCK, and PPP4C were demonstrated to be highly expressed, and SYNJ2 was demonstrated to be lowly expressed in atherosclerosis compared to the control group, which can be diagnostic biomarkers for patients with atherosclerosis (Figure 7A; Supplementary Figure S7).

**Table 4:** The hub genes validated in GEO datasets.

**GEO datasets**	**Hub genes**
Only ICM	SOCS3
Only HF	MRPS21
Only IPH	NAPA GNAI1 YAP1 SMC3 RPS6KA1 PPP1CA FZD6
ICM AND DCM NOT HF NOT IPH	HES1 IL6ST SEC61A1
ICM AND HF NOT DCM NOT IPH	STRN
ICM AND IPH NOT DCM NOT HF	HCK LCP2
ICM AND DCM AND HF NOT IPH	FN1
ICM AND DCM AND IPH NOT HF	GNB2 PPP4C EIF4EBP1 SYNJ2
DCM AND HF AND IPH NOT ICM	MYH10
ICM AND DCM AND HF AND IPH	NET1 FZD7 TBL1XR1

*The hub genes validated in ICM and IPH GEO datasets were labeled blue*.

To validate the correlation between the hub DEMRGs and plaque progression, the MI mice model was applied and ELISA was utilized to investigate the expression of the nine proteins. FZD7, LCP2, HCK, GNB2, and PPP4C were highly expressed and EIF4EBP1 was lowly expressed in the MI group (ApoE-/- and C57Bl/6J). Interestingly, SYNJ2 and NET1 were highly expressed only in ApoE-/- mice model but not in the C57 mice model, which may act as potential therapeutic targets for MI recurrence (Figure 7B). To explore the cardiac function of the hub genes, correlation analysis was used and the protein expressions, including SYNJ2, NET1, FZD7, LCP2, HCK, GNB2, and PPP4C were demonstrated to positively correlate to LVEF, however, that of EIF4EBP1 was demonstrated to negatively correlate to LVEF (Figures 7C–J; Supplementary Figure S8).

## Discussion

The etiology of stable CAD and MI is relatively clear, however, the knowledge of diagnostic biomarkers and treatment targets is still limited (19, 20). MI occurrence can lead to plaque progression by increasing the number of monocytes and macrophages and plaque progression can, in turn, deteriorate MI (4–6). xCell is a recently published method based on Single sample gene set enrichment analysis (ssGSEA) that estimates the abundance scores of 64 immune cell types, including adaptive and innate immune cells, hematopoietic progenitors, epithelial cells, and extracellular matrix cells. It sometimes may not be accurate and calibration parameters should be manually adjusted if the high score from the analysis corresponds to the clearly wrong cell type. In this study, using the xCell immune analysis, we obtained a total of 1,043 DEGs in the GSE41571 dataset and 509 of these DEGs were considered the DEMRGs.

In the event of acute inflammation, a large number of immune cells, especially monocytes, are recruited to the inflammation site to repair the tissue and eventually restore the tissue to homeostasis (21, 22). A network of macrophages can actively take up material, including mitochondria, derived from cardiomyocytes and support mitochondrial homeostasis in the heart (23). Macrophages can also facilitate electrical conduction in the physiological and pathological heart through gap junctions (24–26). However, the specific mechanism of the differentiation of M1-type macrophages to M2-type macrophages and the role of M2-type macrophages has not been fully clarified and needs further studies. In this study, 509 macrophages-related DEGs were screened out using the Venn diagram and further analysis was applied to investigate the potential biomarkers for plaque progression and MI recurrence.

After validation using GEO datasets and atherosclerosis and MI mice model, it was found that the eight proteins, including SYNJ2, NET1, EIF4EBP1, FZD7, LCP2, HCK, GNB2, and PPP4C, can act as diagnostic biomarkers in the deterioration of atherosclerosis and also used as targets to help reduce the mortality of patients. LCP2, EIF4EBP1, HCK, and PPP4C were demonstrated to be highly expressed and SYNJ2 was demonstrated to be lowly expressed in the atherosclerosis mice model. FZD7, LCP2, HCK, GNB2, and PPP4C were highly expressed and EIF4EBP1 was lowly expressed in the MI group (ApoE-/- and C57Bl/6J), while SYNJ2 and NET1 were highly expressed only in ApoE-/- mice model but not in C57 mice model. To investigate the cardiac function, correlation analysis was used and the expression levels of proteins, including SYNJ2, NET1, FZD7, LCP2, HCK, GNB2, and PPP4C were demonstrated to be positively correlated to LVEF, while that of EIF4EBP1 was demonstrated to be negatively correlated to LVEF.

Neuroepithelial cell transforming 1 (NET1) deletion in macrophages decreased RhoA activation, cell adhesion, and motility. Similar to RhoA^−/−^/RhoB^−/−^ macrophages in the mice model, Net1 deletion did not reduce macrophage recruitment to the peritoneum (27). However, Net1 deletion may impair macrophage recruitment in the atherosclerosis mice model, which needs to be investigated. Interestingly, podosome distribution and osteoclastogenesis were affected by the osteoprotegerin-induced inhibition and RANKL-induced promotion of the expression of genes in the Arhgef8/Net1 and RhoGTPase signaling pathway (28, 29). Eukaryotic translation initiation factor 4E binding protein 1 (EIF4EBP1) expression decreased in the MI mice model and Li XQ also reported that the mRNA levels of eIF4E-BP1 and eIF4E decreased in the macrophages pretreated with Lipopolysaccharide (LPS), which was alleviated by Emodin (30). Lymphocyte cytosolic protein 2 (LCP2) is a type of polysaccharide and showed stronger immunomodulatory activity than LCP-1 (31). In the PPI network, LCP2 demonstrated high degrees of connectivity with ZAP70, UBASH3B, and C–C motif chemokine and its receptor, which can regulate T-cell receptor signaling in activated T-lymphocytes (32, 33). “HCK proto-oncogene, Src family tyrosine kinase” (HCK) was identified as a hub gene using weighted correlation network analysis (WGCNA) and validated for the predictive value in identifying future MI-induced heart failure (HF) and in atherosclerosis plaque progression (34, 35). SDF-1 treatment rapidly promoted the hematopoietic Lyn, Fgr, and Hck Src phosphorylation in WT cells (36). Hck tyrosine kinase promoted TNF and IL-6 expression induced by TLR4 and HcK/Fgr kinase deficieny can decrease plaque stability (37, 38). Besides, HCK is also essential for LPS-induced inflammatory response and NLRP3 inflammasome activation (39). G protein subunit beta 2 (GNB2), as a ubiquitin ligase, was lowly expressed through DDX3 knockdown in THP-1 cells (40). LPS inhibited the PKM2 ubiquitination by downregulating the expression of Nedd4L, Nedd4, and Gnb2, which can be rescued by *Lycium barbarum* polysaccharide (41). Gnb2^−/−^ macrophages showed robust Ca2+ transients, decreased cell velocity, and impeded chemokines expression (42). Protein phosphatase 4 catalytic subunit (PPP4C) can interact with cysteine proteinases in *Theileria annulata*, which was involved in ubiquitylation regulating and maturation of spliceosomal snRNPs and further infected macrophages and B lymphocytes, resulting in severe lymphoproliferative diseases (43). Synaptojanin 2 (SYNJ2) was reported as a cell-type-specific methylation biomarker associated with aging, especially in neuronal diseases, such as Alzheimer's disease (44) and progressive hearing loss (45). SYNJ2 knock-down impeded the EGFR endocytic recycling and the formation of cellular lamellipodia and invadopodia and prevented cell migration, suggesting that SYNJ2 may act as a potential druggable target to impair the cell migration (46). Moreover, wake upregulated many genes related to metabolism, including Trio, Synj2, and Gem, which were involved in the elongation of peripheral astrocytic processes (47). However, the effects of SYNJ2 on macrophages in plaque progression and cardiac neurophysiopathology are unclear and need to be more investigated. Frizzled class receptor 7 (FZD7), as the membrane receptor of Wnt/b-catenin signal pathway, controlled both vaso-obliteration and neovascular phases in oxygen-induced retinopathy and single intravitreal microinjection of a monoclonal antibody against Fzd7 or a soluble Fzd7 receptor reduced retinal neovascularization (48). Wnt5a/FZD7 can regulate ABCG2 expression and YTHDF1 promoted translation of FZD7 in an m6A-dependent manner and mutated YTHDF1 enhanced FZD7 expression, resulting in the Wnt/β-catenin pathway over-activation (49, 50). FZD7-mediated PCP signaling increased YAP expression by activating RhoA, which may also act as a potential target for macrophages in CVD (51). Besides, epicardial erythropoietin treatment enhanced FOS and Fzd7 expression and therefore, increased cardiac repaired critical indicators expressions, such as SDF-1, CXCR4, CD34, Bcl-2, cyclin D1, and MMP2, which elevated intracardiac mesenchymal proliferation during early MI period (52).

There are some limitations. At first, only hub DEMRGs were validated in GEO datasets and atherosclerosis and MI mice model. The other neighbor DEMRGs are still needed to be investigated. Second, the sample sizes were not too large, however, after validation, the results are highly reliable.

## Conclusion

Based on our current study, our research provided bioinformatics analysis of macrophages-related genes biomarkers in the deterioration of atherosclerosis. The screened hub DEMRGs, SYNJ2, NET1, FZD7, LCP2, HCK, GNB2, EIF4EBP1, and PPP4C, may act as therapeutic targets for treatment and prediction for the patients with plaque progression and MI recurrent events. The kit of the eight hub DEMRGs may test plaque progression and MI recurrent events and help diagnose and treat MI-induced HF, thus decreasing mortality and morbidity.

## Data Availability Statement

Publicly available datasets were analyzed in this study. This data can be found here: The data, including GSE41571, GSE163154, GSE135055, and GSE116250 were downloaded from the Gene Expression Omnibus (GEO) dataset.

## Ethics Statement

The animal study was reviewed and approved by Protocols were approved by Institute of Radiation Medicine, the Chinese Academy of Medical Science, which conform to the Guide for the Care and Use of Laboratory Animals.

## Author Contributions

YZ, YL, and BQ downloaded and analyzed the datasets. YZ, YL, YW, WG, and ZQ carried out the PPI analysis and investigated the hub genes. YZ validated the results in the mice model. YZ, XC, ZQ, and TL wrote and supervised the study. All authors contributed to the article and approved the submitted version.

## Funding

This work was funded by Tianjin Key Medical Discipline (Specialty) Construction Project, the Tianjin “Project + Team” Key Training Special Project, China (no. XC202040), the Tianjin “131” Innovative Talent Team Project (no. 201939), Key Project of Tianjin Natural Science Foundation (no. 21JCZDJC00240), the Tianjin Municipal Health and Health Committee Science and Technology Project (no. ZD20001), Tianjin Health Committee traditional Chinese medicine and integrated traditional Chinese and Western medicine project (no. 2021139), and the Tianjin Municipal Health and Health Committee Science and Technology Talent Cultivation Project (no. KJ20008).

## Conflict of Interest

The authors declare that the research was conducted in the absence of any commercial or financial relationships that could be construed as a potential conflict of interest.

## Publisher's Note

All claims expressed in this article are solely those of the authors and do not necessarily represent those of their affiliated organizations, or those of the publisher, the editors and the reviewers. Any product that may be evaluated in this article, or claim that may be made by its manufacturer, is not guaranteed or endorsed by the publisher.

## Abbreviations

CAD: Coronary artery disease
MI: Myocardial infarction
STEMI: ST-segment elevation myocardial infarction
DEMRGs: Differentially expressed macrophages-related genes
HF: Heart failure
GEO: Gene Expression Omnibus
PCC: Pearson correlation coefficients
DEGs: Differentially expressed genes
GO: Gene Ontology
KEGG: Kyoto Encyclopedia of Genes and Genomes
GSEA: Gene Set Enrichment Analysis
PPI: Protein-protein internet
TF: Transcription factor
CTD: Comparative Toxicogenomics Database
ROC: Receiver operation characteristic
AUC: Area under curve
ECG: Electrocardiogram
LVEF: Left ventricular ejection fraction
LVFS: Left ventricular fraction shorten
IPH: Intraplaque hemorrhage
DCM: Dilated cardiomyopathy
ICM: Ischemic cardiomyopathy.
