# Supplementary material for: Macrophages-Related Genes Biomarkers in the Deterioration of Atherosclerosis
Source: Front Cardiovasc Med. 2022 Jun 30;9:890321. doi: 10.3389/fcvm.2022.890321 (PMC9282674; doi:10.3389/fcvm.2022.890321)
Supplement: Supplementary file 1 [file Data_Sheet_1.docx]

Supplementary Table 1. The detailed information for the datasets for validation of hub genes.

| Reference | Sample | GEO | Experimental cohort | Control cohort | Platform |
| --- | --- | --- | --- | --- | --- |
| Jin H et al. | carotid plaque | GSE163154 | 27 intraplaque haemorrhage | 16 no intraplaque haemorrhage | GPL6104 |
| Yamaguchi T et al. | left ventricular heart tissue | GSE116250 | 37 dilated cardiomyopathy patients | 14 healthy donors | GPL16791 |
| Yamaguchi T et al. | left ventricular heart tissue | GSE116250 | 13 ischemic cardiomyopathy patients | 14 healthy donors | GPL16791 |
| Hua X et al. | left ventricular heart tissue | GSE135055 | 21 heart failure patients | 9 healthy donors | GPL16791 |

Supplementary Table 2. The description of screened hub genes.

| Degree | Degree | Expr. | logFC | full name |
| --- | --- | --- | --- | --- |
| GNAI1 | 113 | 204525.9 | -2.385810199 | G protein subunit alpha i1 |
| MRPS2 | 56 | 75908.13 | -2.129496281 | mitochondrial ribosomal protein S28 |
| YAP1 | 51 | 171932.4 | 1.087706651 | [Yes1 associated transcriptional regulator](https://www.ncbi.nlm.nih.gov/gene/10413) |
| UBC | 50 | 533809.4 | 1.087706651 | ubiquitin C |
| HCK | 45 | 79689.21 | -2.385810199 | "HCK proto-oncogene, Src family tyrosine kinase" |
| SMC3 | 44 | 62813.87 | -2.385810199 | structural maintenance of chromosomes 3 |
| SEC61A1 | 43 | 99786.08 | 1.087706651 | [SEC61 translocon subunit alpha 1](https://www.ncbi.nlm.nih.gov/gene/29927) |
| ERBB4 | 42 | 109371.4 | -2.385810199 | [erb-b2 receptor tyrosine kinase 4](https://www.ncbi.nlm.nih.gov/gene/2066) |
| GNB2 | 41 | 43454.49 | 1.287542458 | G protein subunit beta 2 |
| PPP1CA | 40 | 160832.4 | 1.087706651 | protein phosphatase 1 catalytic subunit alpha |
| SOCS3 | 40 | 47229.24 | -2.385810199 | suppressor of cytokine signaling 3 |
| FGFR1 | 36 | 54645.42 | -2.385810199 | [fibroblast growth factor receptor 1](https://www.ncbi.nlm.nih.gov/gene/2260) |
| STRN | 31 | 37887.54 | -2.385810199 | striatin |
| FN1 | 30 | 56463.01 | -2.385810199 | [fibronectin 1](https://www.ncbi.nlm.nih.gov/gene/2335) |
| RPS6KA1 | 30 | 85581.33 | 1.087706651 | ribosomal protein S6 kinase A1 |
| FLT1 | 29 | 43449.44 | -2.385810199 | [fms related receptor tyrosine kinase 1](https://www.ncbi.nlm.nih.gov/gene/2321) |
| SYNJ2 | 29 | 27772.1 | -2.385810199 | [synaptojanin 2](https://www.ncbi.nlm.nih.gov/gene/8871) |
| PPP4C | 28 | 25275.8 | 1.087706651 | protein phosphatase 4 catalytic subunit |
| NET1 | 28 | 46191.78 | 1.087706651 | neuroepithelial cell transforming 1 |
| FZD6 | 27 | 10063.42 | -2.385810199 | frizzled class receptor 6 |
| NAPA | 25 | 47209.5 | -2.129496281 | NSF attachment protein alpha |
| FZD7 | 25 | 32604.36 | -2.385810199 | frizzled class receptor 7 |
| LCP2 | 24 | 21266.03 | -2.385810199 | lymphocyte cytosolic protein 2 |
| EIF4EBP1 | 24 | 58657.63 | -2.385810199 | eukaryotic translation initiation factor 4E binding protein 1 |
| HES1 | 22 | 27947.23 | 1.287542458 | [hes family bHLH transcription factor 1](https://www.ncbi.nlm.nih.gov/gene/3280) |
| MYH10 | 21 | 28339.74 | -2.129496281 | myosin heavy chain 10 |
| IL6ST | 21 | 20883.83 | -2.385810199 | interleukin 6 signal transducer |
| TBL1XR1 | 21 | 30504.57 | -2.385810199 | TBL1X receptor 1 |
