# Supplementary figures and images for: Macrophages-Related Genes Biomarkers in the Deterioration of Atherosclerosis
Source: Front Cardiovasc Med. 2022 Jun 30;9:890321. doi: 10.3389/fcvm.2022.890321 (PMC9282674; doi:10.3389/fcvm.2022.890321)

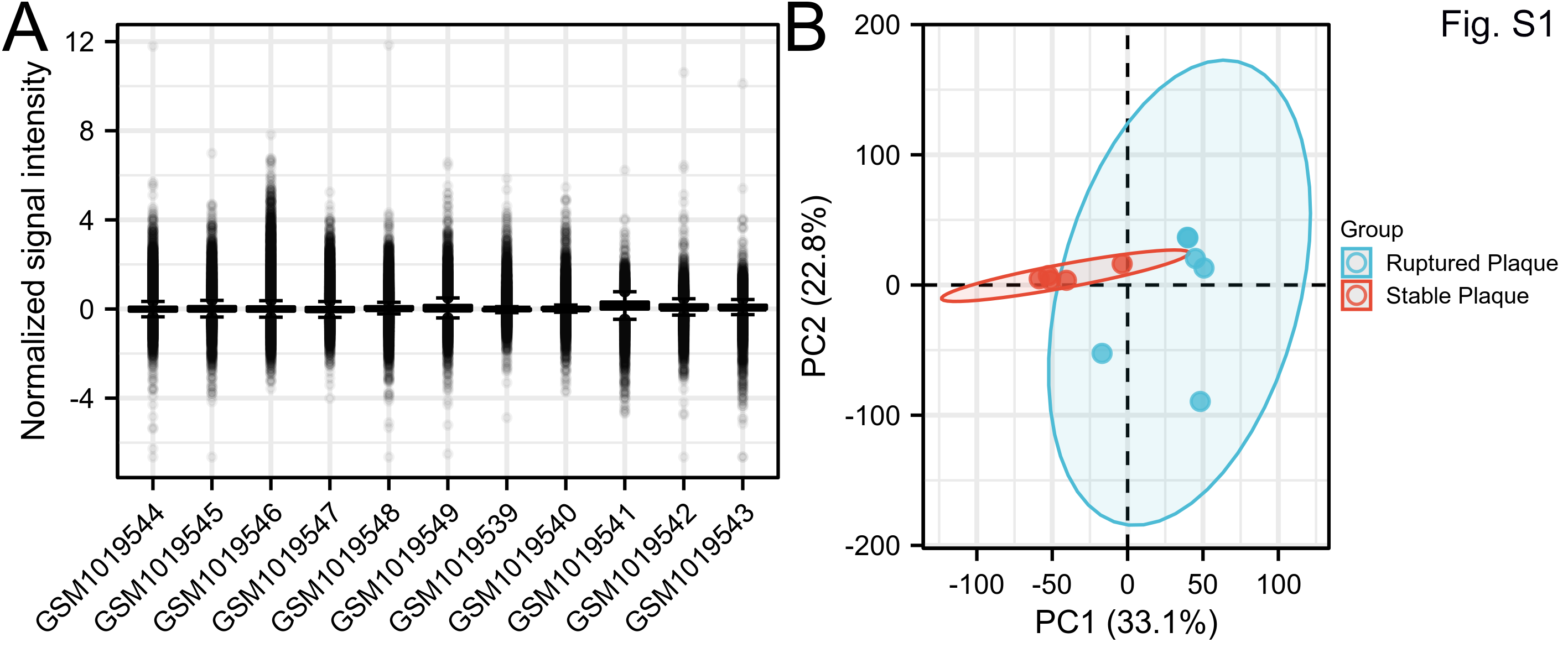

Supplement: Supplementary file 2 [file Image_1.TIFF]

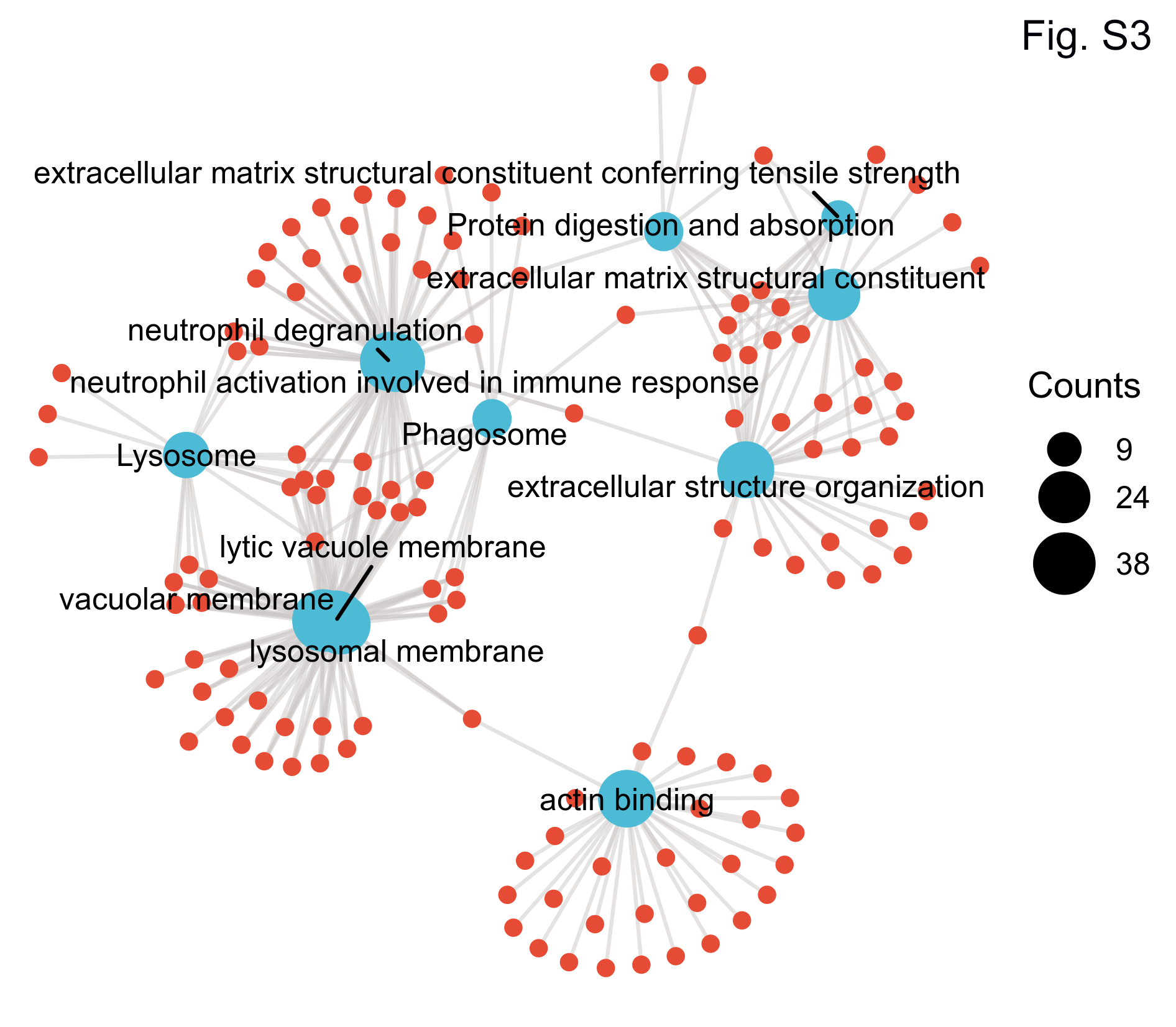

Supplement: Supplementary file 4 [file Image_3.TIFF]

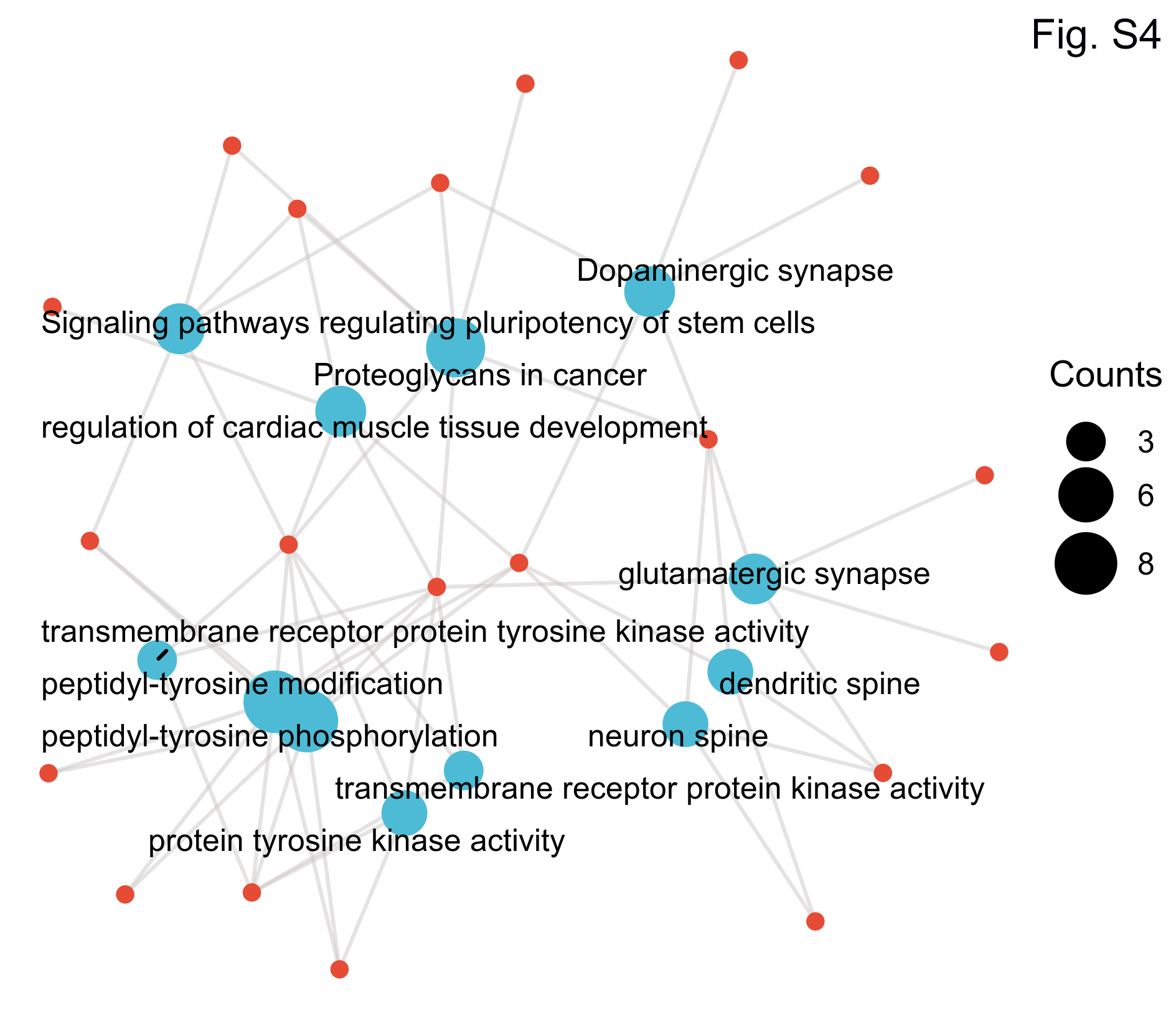

Supplement: Supplementary file 5 [file Image_4.TIFF]

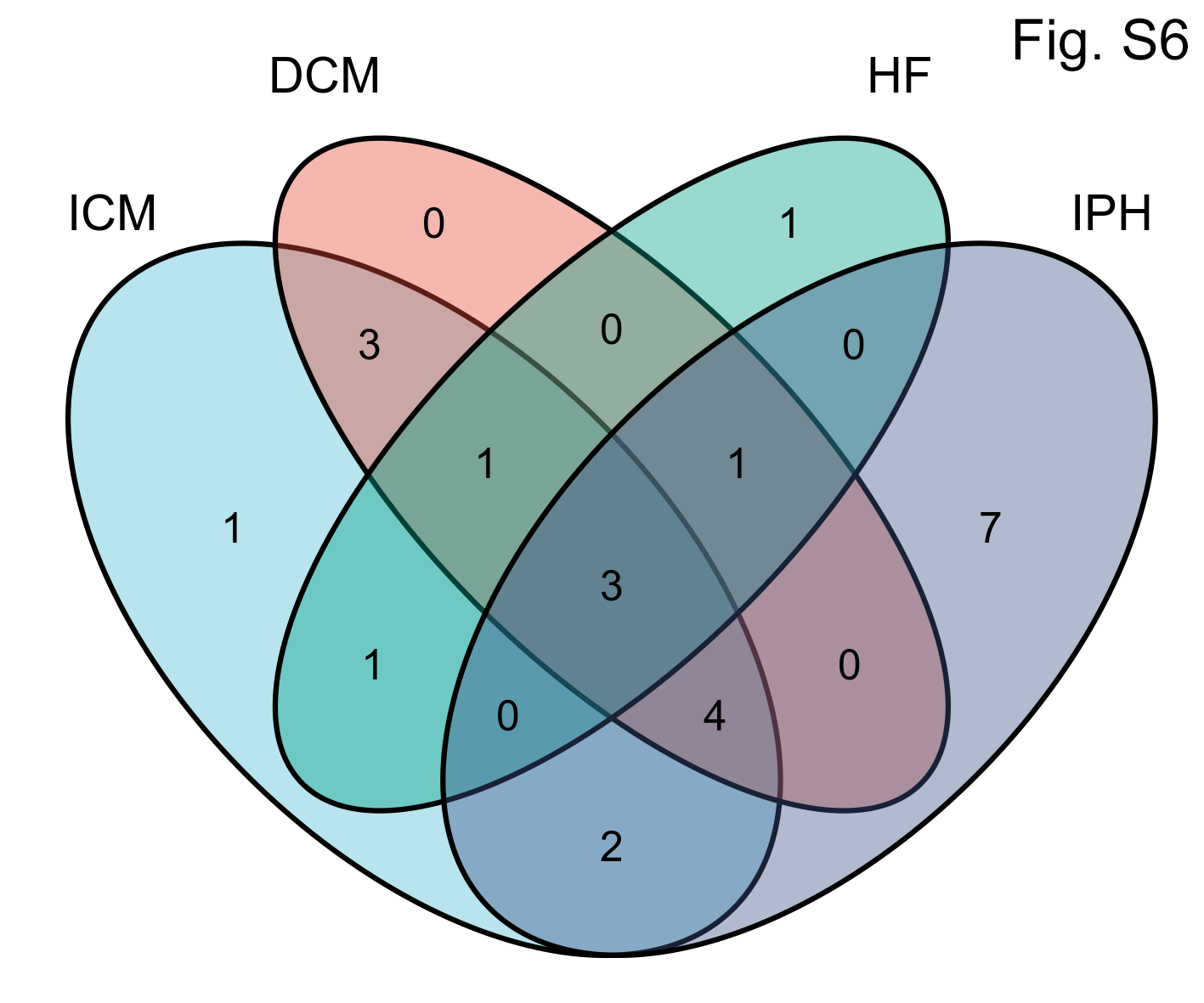

Supplement: Supplementary file 7 [file Image_6.TIFF]

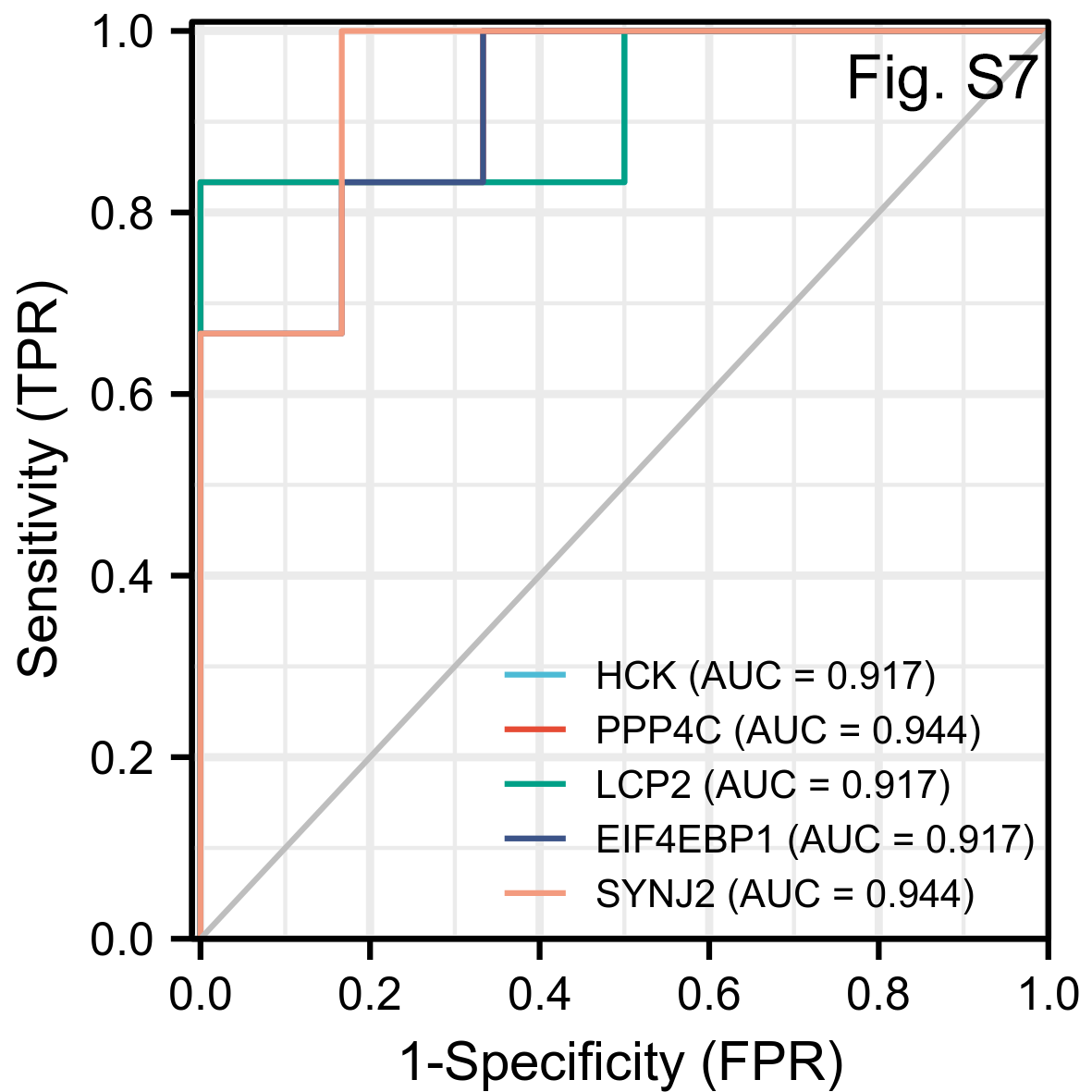

Supplement: Supplementary file 8 [file Image_7.TIFF]

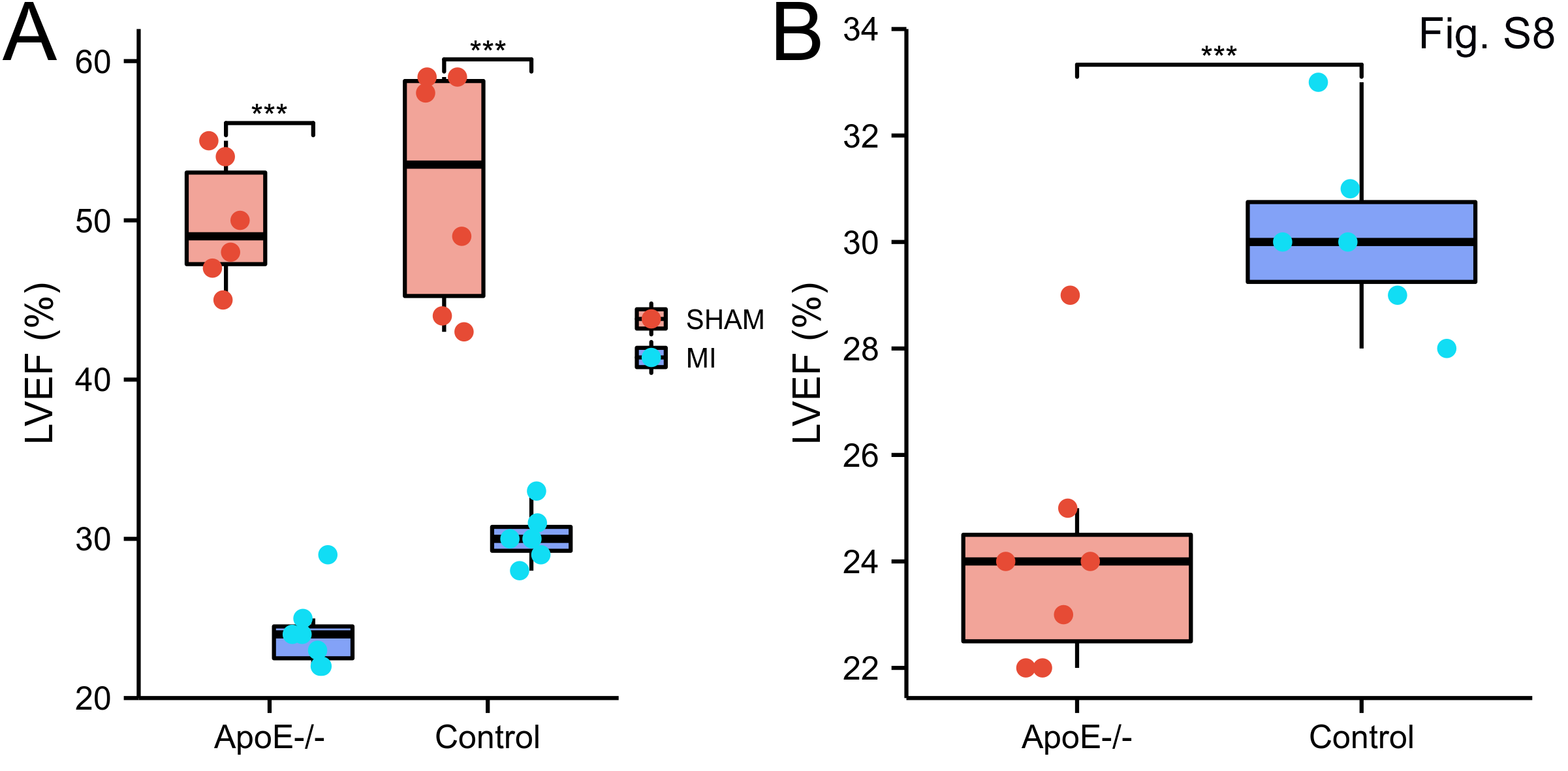

Supplement: Supplementary file 9 [file Image_8.TIFF]
